# Supplementary material for: Differential Expression Analysis Reveals Possible New Quaternary Ammonium Compound Resistance Gene in Highly Resistant Serratia sp. HRI
Source: Microorganisms. 2024 Sep 13;12(9):1891. doi: 10.3390/microorganisms12091891 (PMC11433835; doi:10.3390/microorganisms12091891)
Supplement: Supplementary file 1 [file microorganisms-12-01891-s001.zip › microorganisms-3201553-supplementary.pdf]

Supplementary materials

Tables S1-S4 detail the most differentially expressed genes in *Serratia* sp. HRI after treatment with BC at four different timepoints. These represent novel gene targets in the study of QAC-resistance.

Table S1. Greatest differentially expressed genes of untreated and BC treated samples at 10 minutes exposure time (Log Fold change > 3 and Z value >3).

	Comparison/Gene	+10 vs D10
1.	Putative plasmid stabilization protein	13.4760753213
2.	Fimbrial protein	10.9440165305
3.	Conjugative transfer protein TrbG	14.5352936046
4.	Conjugative transfer protein TrbF	14.5352936046
5.	(4S)-4-hydroxy-5-phosphonooxypentane-2,3-dione isomerase (EC 5.3.1.32)	-12.5601106803
6.	Type IV pilin PilA	12.3082081804
7.	Fimbrial protein precursor	13.0711237722
8.	Sigma-fimbriae uncharacterized paralogous subunit	13.249720869
9.	Alkanesulfonate ABC transporter substrate-binding protein SsuA	12.5887989465
10.	Membrane fusion component of MSF-type tripartite multidrug efflux system	11.2712879329
11.	Mg(2+)-transport-ATPase-associated protein MgtC	13.8327614789
12.	Outer membrane component of tripartite multidrug resistance system	11.2712879329
13.	Cation transport regulator chaB	-13.8720207102
14.	Histone acetyltransferase HPA2 and related acetyltransferases	13.3420607745
15.	Mg(2+)-transport-ATPase-associated protein MgtC	13.1663419959
16.	Tripartite tricarboxylate transporter TctB family	12.5552481776
17.	Transcriptional regulator, TetR family	-13.184153195
18.	ABC transporter, substrate-binding protein (cluster 3, basic aa/glutamine/opines)	13.5870625536
19.	Phosphonate ABC transporter ATP-binding protein PhnC (TC 3.A.1.9.1)	13.4933926521
20.	Maleamate amidohydrolase, NicF (EC 3.5.1.107)	14.0162424481
21.	Putative membrane protein	11.9569568939
22.	ABC transporter, permease protein 1 (cluster 4, leucine/isoleucine/valine/benzoate)	13.5657566182
23.	Alkanesulfonate utilization operon LysR-family regulator Cbl	11.24911938
24.	LSU ribosomal protein L36p @ LSU ribosomal protein L36p, zinc-independent	-14.3539944907
25.	1,2-phenylacetyl-CoA epoxidase, subunit C (EC 1.14.13.149)	13.24556459
26.	Ureidoglycolate dehydrogenase (EC 1.1.1.154)	13.5230718269
27.	Maleate cis-trans isomerase (EC 5.2.1.1)	13.1492199823
28.	Glutathione S-transferase, unnamed subgroup (EC 2.5.1.18)	13.4814462813
29.	Ribosome modulation factor	-12.9046891827
30.	1,2-epoxyphenylacetyl-CoA isomerase (EC 5.3.3.18)	13.8628794652
31.	FMN reductase (NADPH) (EC 1.5.1.38)	11.3489272855
32.	1,2-phenylacetyl-CoA epoxidase, subunit D (EC 1.14.13.149)	13.7946392393
33.	Alpha-D-ribose 1-methylphosphonate 5-triphosphate synthase subunit PhnG (EC 2.7.8.37)	13.3128546002
34.	Omega-amino acid--pyruvate aminotransferase (EC 2.6.1.18)	13.5698674726
35.	Antibiotic biosynthesis monooxygenase	13.5905285737

	Comparison/Gene	+10 vs D10
36.	Cold shock protein of CSP family => CspD (naming convention as in E.coli)	-13.8721939018
37.	Uncharacterized protein YobF	-13.7876067276
38.	Cold shock protein of CSP family => CspE (naming convention as in E.coli)	-16.5513598005
39.	PTS system, glucitol/sorbitol-specific IIC component	11.2735158897
40.	Acid shock protein precursor	11.3123894964
41.	Methionine sulfoxide reductase-associated methionine-rich protein	-12.7355835237
42.	Uncharacterized protein YbaM	-13.3461951305
43.	Hypothetical protein	11.321127628
44.	UPF0380 proteins YafZ and homologs	12.7078896909
45.	Hypothetical protein	-12.0286036832
46.	Hypothetical protein	13.6786551524
47.	Hypothetical protein	13.4480388501
48.	Hypothetical protein	13.9057536497
49.	FIG01055767: hypothetical protein	-12.1464827606
50.	Hypothetical protein	-12.7207829235
51.	Hypothetical protein	11.925272549
52.	Hypothetical protein	-12.6074459097
53.	Hypothetical protein	-13.1347054624
54.	Hypothetical protein	-15.2665742779
55.	Hypothetical protein	-11.7604413599
56.	Hypothetical protein	-12.6743489783
57.	Hypothetical protein	13.249720869
58.	UPF0758 family protein	12.6180803198
59.	Hypothetical protein	13.6786551524
60.	Hypothetical protein	-12.1266786641
61.	Uncharacterized protein KOX_20805	-11.9573052237
62.	Hypothetical protein	-11.9701274661
63.	Hypothetical protein	-12.7381154878
64.	Hypothetical protein	-13.8720207102
65.	Hypothetical protein	-12.4444092836
66.	Yts1S protein	13.2690696834
67.	FIG01056587: hypothetical protein	-13.0250443717
68.	Uncharacterized proteins YbdD and YjiX	-14.1001038107

Table S2. Greatest differentially expressed genes of untreated and BC treated samples at 20 minutes exposure time (Log Fold change > 3 and Z value >3).

	Comparison/Gene	+20 vs D20
1.	tyrosine-type recombinase/integrase	-13.5289180038
2.	Uncharacterized MFS-type transporter	-12.4740785499
3.	Putative outer membrane lipoprotein PANA_1770	-12.2852172461
4.	Osmotically-inducible lipoprotein OsmB	12.6401477589
5.	UPF0391 membrane protein YtjA	-12.5757070473
6.	Alpha-D-ribose 1-methylphosphonate 5-triphosphate synthase subunit PhnG (EC 2.7.8.37)	13.6598621382
7.	Triphosphoribosyl-dephospho-CoA synthase (EC 2.4.2.52)	14.7535353858
8.	Citrate lyase holo-[acyl-carrier-protein] synthase (EC 2.7.7.61)	14.7535353858
9.	Fructose-6-phosphate aldolase	11.7094500417
10.	PTS system N-acetylgalactosamine-specific IIB component	12.8263558238
11.	PTS-regulatory domain-containing protein YhfY	10.7091269032

	Comparison/Gene	+20 vs D20
12.	Glycerate kinase (EC 2.7.1.31)	9.22323
13.	Trimethylamine-N-oxide reductase (EC 1.7.2.3) TorZ	13.2274204023
14.	4-hydroxy-2-oxoglutarate aldolase (EC 4.1.3.16) @ 2-dehydro-3-deoxyphosphogluconate aldolase (EC 4.1.2.14)	12.8235821951
15.	2-keto-3-deoxy-L-fuconate dehydrogenase	13.8063683675
16.	dTDP-glucose 46-dehydratase (EC 4.2.1.46)	-10.4850443657
17.	Uncharacterized protein YbaM	-12.5211881822
18.	Uncharacterized protein YaiN in in formaldehyde detoxification operon	11.3484736687
19.	hypothetical protein	-14.0993395908
20.	hypothetical protein	11.0901852144
21.	hypothetical protein	-11.8936731552
22.	hypothetical protein	-11.4707554572
23.	hypothetical protein	-11.5548574135
24.	hypothetical protein	-12.4951305002
25.	hypothetical protein	-12.6179495075
26.	UPF0380 proteins YafZ and homologs	12.4209497168
27.	hypothetical protein	12.4929121867
28.	hypothetical protein	-11.3250805436
29.	FIG01055767: hypothetical protein	-11.8992171787
30.	hypothetical protein	-12.2500437325
31.	UPF0370 protein YpfN	-13.9312268987
32.	hypothetical protein	-13.5595921881
33.	hypothetical protein	-12.8829830513
34.	hypothetical protein	-14.2291342455
35.	hypothetical protein	-12.9085239736
36.	hypothetical protein	-12.1003601035

Table S3. Greatest differentially expressed genes of untreated and BC treated samples at 30 minutes exposure time (Log Fold change > 3 and Z value >3).

	Comparison/Gene	+30 vs D30
1.	putative plasmid stabilization protein	10.6620897575
2.	putative plasmid stabilization protein	6.40066
3.	MrfE	11.2179819243
4.	UPF0386 protein YjhX	11.5988527412
5.	[4Fe-4S] cluster carrier protein NfuA	-5.28795
6.	Fructose-6-phosphate aldolase	10.6024297843
7.	ABC transporter permease protein (cluster 2 ribose/xylose/arabinose/galactose)	10.6423044584
8.	L-threonate/D-erythronate proton symporter	11.3536430802
9.	Putative outer membrane lipoprotein PANA_1770	11.4805933188
10.	ABC-type nitrate/sulfonate/bicarbonate transport system ATPase component	11.1306992542
11.	Glutathione S-transferase unnamed subgroup (EC 2.5.1.18)	10.6629709448
12.	Copper resistance inner membrane protein PcoD	11.2903644992
13.	Inner membrane protein YfeZ	10.6309496268
14.	Protein secretion chaperonin CsaA	11.4270980478
15.	Biofilm PGA outer membrane secretin PgaA	6.14447
16.	Guanidinobutyrase (EC 3.5.3.7)	11.2383151652
17.	Urea ABC transporter ATPase protein UrtE	10.8019265652

	Comparison/Gene	+30 vs D30
18.	Asparagine synthetase [glutamine-hydrolyzing] (EC 6.3.5.4)	5.97921
19.	phenylacetyl-CoA epoxidase subunit D (EC 1.14.13.149)	11.2184966394
20.	PTS system N-acetylgalactosamine-specific IIA component @ PTS system galactosamine-specific IIA component	11.0939588861
21.	Aspartate--ammonia ligase (EC 6.3.1.1)	6.67042
22.	Histone acetyltransferase HPA2 and related acetyltransferases	11.9407723967
23.	Copper-binding protein PcoE	10.5178563556
24.	Antibiotic biosynthesis monooxygenase	10.9940917478
25.	N-acetyl-D-glucosamine kinase (EC 2.7.1.59)	11.3798115438
26.	Transcriptional regulator HxlR family	10.661653415
27.	Osmotically-inducible lipoprotein OsmB	11.1653090156
28.	Osmoprotectant ABC transporter permease protein OsmY	11.0071394254
29.	Oxidoreductase short-chain dehydrogenase/reductase family	11.31040261
30.	FIG01056587: hypothetical protein	11.6458565417
31.	Uncharacterized protein YjdI	11.4387190923
32.	L-threonate/D-erythronate transcriptional regulator DeoR family	10.1832135517
33.	hypothetical protein	11.5007871178
34.	hypothetical protein	10.8097199041
35.	hypothetical protein	10.2258340355
36.	UPF0758 family protein	10.0520381502
37.	hypothetical protein	10.4017541784
38.	UPF0380 proteins YafZ and homologs	10.0270213146
39.	hypothetical protein	12.0114998319
40.	hypothetical protein	11.8327120392
41.	Probable exported protein YPO2521	10.0704429986
42.	hypothetical protein	12.4062342418
43.	hypothetical protein	10.4230213385
44.	hypothetical protein	10.4841381312
45.	Corresponds to STY4575 from Accession AL513382: Salmonella typhi CT18	12.1015161408
46.	hypothetical protein	11.0859968595
47.	hypothetical protein	10.4017541784
48.	hypothetical protein	10.9921665215

Table S4. Greatest differentially expressed genes of untreated and BC treated samples at 90 minutes exposure time (Log Fold change > 3 and Z value >3).

	Comparison/Gene	+90 vs D90
1.	putative plasmid stabilization protein	11.036235441
2.	Fimbrial protein	11.2816229146
3.	Conjugative transfer protein TrbC	9.57989
4.	T6SS component TssB (ImpB/VipA)	-7.24162
5.	T6SS component Hcp	-7.5119
6.	Sigma-fimbriae uncharacterized subunit	11.6158732838
7.	Sigma-fimbriae uncharacterized paralogous subunit	11.6158732838
8.	Transposase and inactivated derivatives	-11.1819286925
9.	Coupling protein VirD4 ATPase required for T-DNA transfer	9.57989
10.	Conjugative transfer protein TrbD	9.57989
11.	Conjugative transfer protein TrbB	9.57989
12.	Fimbrial protein precursor	11.1567214635
13.	Oligopeptide ABC transporter permease protein OppB (TC 3.A.1.5.1)	10.329426369
14.	OutS lipoprotein precursor	12.8530599866

	Comparison/Gene	+90 vs D90
15.	Inner membrane protein YfdC	10.9867166791
16.	putative membrane protein	13.0501275343
17.	ABC transporter permease protein 1 (cluster 4 leucine/isoleucine/valine/benzoate)	10.9381534447
18.	glutamine synthetase family protein	11.513890408
19.	Citrate lyase holo-[acyl-carrier-protein] synthase (EC 2.7.7.61)	12.3058508537
20.	Guanidinobutyrase (EC 3.5.3.7)	11.4576117329
21.	PTS system N N'-diacetylchitobiose-specific IIB component (EC 2.7.1.196)	-13.3633819148
22.	Alpha-D-ribose 1-methylphosphonate 5-triphosphate synthase subunit PhnG (EC 2.7.8.37)	11.6205497561
23.	Triphosphoribosyl-dephospho-CoA synthase (EC 2.4.2.52)	12.3058508537
24.	CopG domain-containing protein	9.57989
25.	1,2-phenylacetyl-CoA epoxidase subunit D (EC 1.14.13.149)	10.8021285786
26.	Histone acetyltransferase HPA2 and related acetyltransferases	10.8841247185
27.	ilvB operon leader peptide IvbL	13.8116054902
28.	Glucitol operon activator protein	12.9598480219
29.	1,2-phenylacetyl-CoA epoxidase subunit C (EC 1.14.13.149)	11.2514646579
30.	Maleate cis-trans isomerase (EC 5.2.1.1)	10.504451565
31.	Copper-binding protein PcoE	10.9519621854
32.	23-dehydroadipyl-CoA hydratase (EC 4.2.1.17)	11.6960372965
33.	Transcriptional regulator GntR family	11.284378791
34.	NAD-specific glutamate dehydrogenase (EC 1.4.1.2); NADP-specific glutamate dehydrogenase (EC 1.4.1.4)	-6.66689
35.	Methionine sulfoxide reductase cytochrome b subunit	10.6217497007
36.	Alkaline proteinase inhibitor precursor	11.3151835343
37.	Osmotically-inducible lipoprotein OsmB	11.2071119612
38.	Uncharacterized protein YaaX/YpeC	11.9294063698
39.	YciY family protein	12.7127096901
40.	hypothetical protein	13.7978641373
41.	hypothetical protein	13.4120405146
42.	hypothetical protein	12.1073217097
43.	hypothetical protein	-12.9781849021
44.	hypothetical protein	11.3685828503
45.	Uncharacterized protein STM4440	-12.3271680613
46.	hypothetical protein	12.5286396859
47.	DUF2618 domain-containing protein	-13.3097463365
48.	hypothetical protein	-12.6841590635
49.	hypothetical protein	12.3055231423
50.	UPF0380 proteins YafZ and homologs	10.6224545461
51.	hypothetical protein	12.44712198
52.	hypothetical protein	-12.8804884788
53.	hypothetical protein	11.1055091593
54.	hypothetical protein	10.9134130412
55.	hypothetical protein	10.4869255013
56.	hypothetical protein	11.1771890372
57.	hypothetical protein	10.3649663183
58.	hypothetical protein	11.1710141256
59.	hypothetical protein	12.5635109037
60.	FIG01055513: hypothetical protein	11.6858131574
61.	hypothetical protein	11.4472537352

Comparison/Gene	+90 vs D90
62. hypothetical protein	11.1771890372
63. putative TonB-dependent receptor	12.1983959276
64. hypothetical protein	12.5286396859

Figures S1-S3 detail real-time PCR amplification plots, standard curve plots, and melt curve plots. Table S5 depicts qPCR raw data.

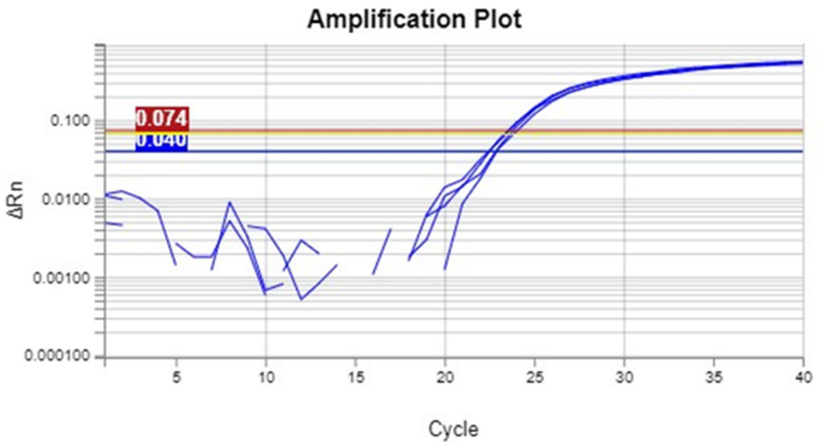

Figure S1. Primer optimisation for the uncharacterised MFS efflux pump by gradient PCR at four different temperatures, 56°C, 58°C, 60°C and 62°C.

(a)

(b)

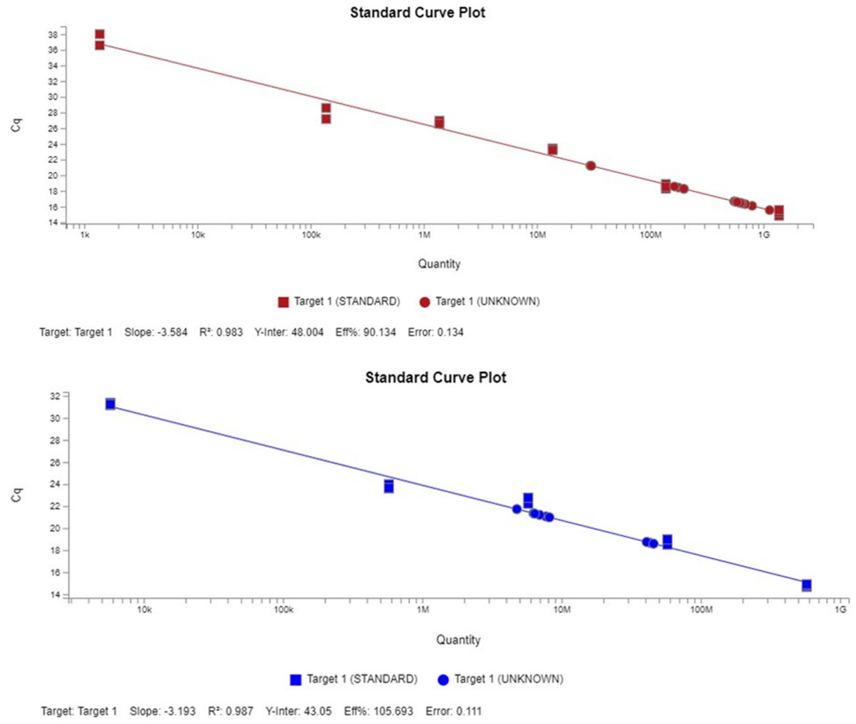

(c)

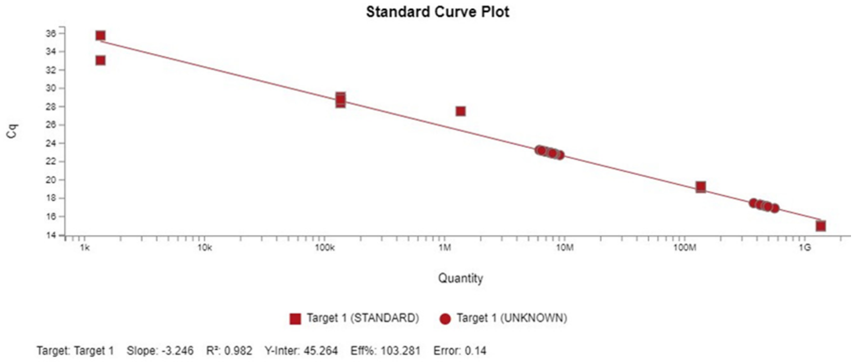

Figure S2. Standard curves generated after real-time PCR amplification of the UMFS1 gene including standards and samples for three biological replicates (a), (b), (c).

(a)

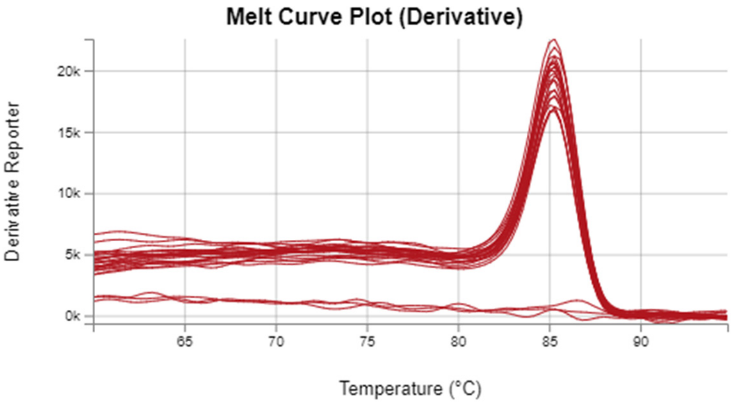

(b)

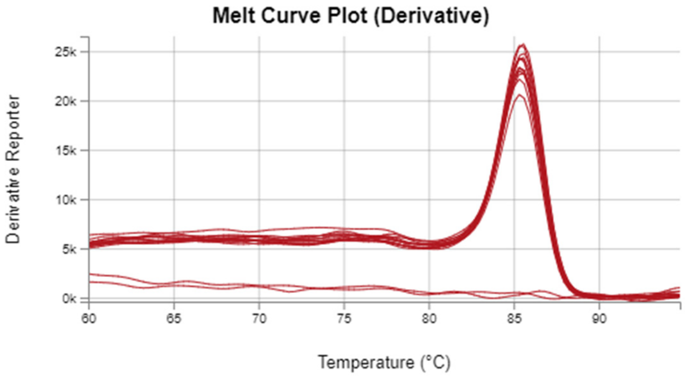

(c)

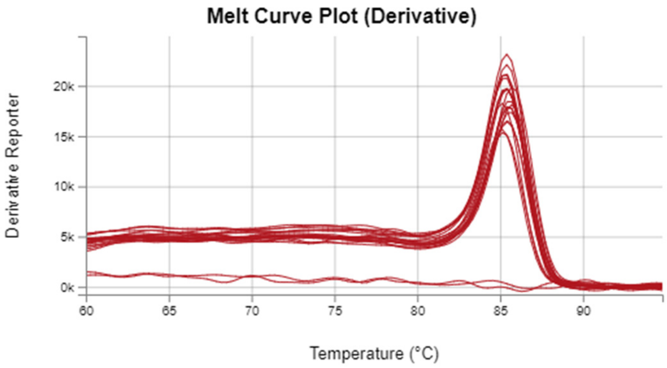

Figure S3. Dissociation (melt) curves generated after real-time PCR amplification of the UMFS1 gene for all standards, samples and negative controls.

Table S4. Expression and gene count data for all samples. Where no amplification signal was detected, the software output stated "undetermined".

Sample	Untreated	Cq	BC 20	Cq	BC 90	Cq	DDAC	Cq	Virukill	Cq
A	2603500	23,2	20474627	17,0	1281506	21,2	8193591	18,2	24862733	16,5
B	3524865	22,9	17892098	17,2	1267501	21,2	7313332	18,4	22858583	16,6
C	2717790	23,1	18972673	17,1	1264176	21,2	8146890	18,3	25147691	16,5
D	2893892	23,0	15895435	17,4	1285377	21,2	6752769	18,6	23628563	16,5
E	3209768	23,0	17989960	17,2	773002	21,0	629570	21,3	4299274	18,7
F	3531666	22,8	23777856	16,8	818065	20,9	643403	21,3	4086851	18,7
G	3421268	22,9	20061651	17,1	696184	21,2	478014	21,4	4578795	18,6
H	3001536	23,0	20934518	17,0	1374809	20,8	869951	21,4	7075109	18,4
I	2901286	23,0	18972673	17,1	957955	21,3	1096348	21,2	6978882	18,4
J	3246866	22,9			1095585	21,1	1101246	21,2	6791275	18,4
K	3228535	22,9			1092385	21,1	982351	21,2	7268119	18,3
L	3353241	22,8			1294181	20,9				
M	3857644	22,6								
Negative	Undetermined		Undetermined		Undetermined		Undetermined		Undetermined	
Neg RT	Undetermined		Undetermined		Undetermined		Undetermined		Undetermined	
Ave	3191681	22,93	19441276	17,10	1100060	21,09	3291587	20,23	12506897	17,78
